# Supplementary material for: Evidence supported by Mendelian randomization: impact on inflammatory factors in knee osteoarthritis
Source: Front Med (Lausanne). 2024 May 28;11:1382836. doi: 10.3389/fmed.2024.1382836 (PMC11165061; doi:10.3389/fmed.2024.1382836)
Supplement: Supplementary file 1 [file Data_Sheet_1.ZIP › data/GCST90274844_5e6/844.forest.pdf]

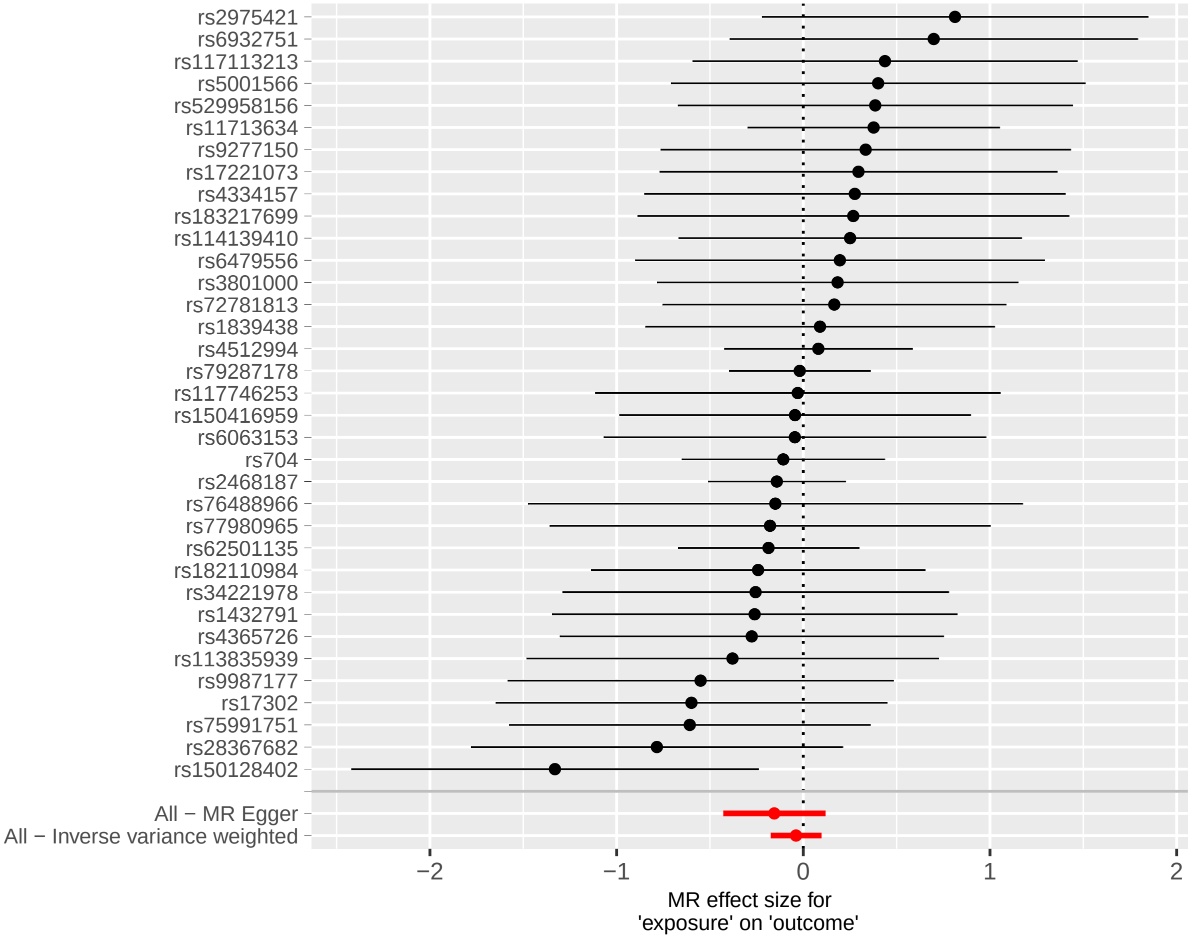
rs2975421
rs6932751
rs117113213
rs5001566
rs529958156
rs11713634
rs9277150
rs17221073
rs4334157
rs183217699
rs114139410
rs6479556
rs3801000
rs72781813
rs1839438
rs4512994
rs79287178
rs117746253
rs150416959
rs6063153
rs704
rs2468187
rs76488966
rs77980965
rs62501135
rs182110984
rs34221978
rs1432791
rs4365726
rs113835939
rs9987177
rs17302
rs75991751
rs28367682
rs150128402
All − MR Egger
All − Inverse variance weighted
−2
−1
0
1
2
MR effect size for
'exposure' on 'outcome'
